# Sibling mortality burden in low-income countries: A descriptive analysis of sibling death in Africa, Asia, and Latin America and the Caribbean

**DOI:** 10.1371/journal.pone.0236498

**Published:** 2020-10-14

**Authors:** Emily Smith-Greenaway, Abigail Weitzman

**Affiliations:** 1 Department of Sociology, University of Southern California, Los Angeles, CA, United States of America; 2 Department of Sociology, University of Texas at Austin, Austin, TX, United States of America; University of Louvain, BELGIUM

## Abstract

In high-income countries, emerging research suggests sibling bereavement can have significant health and life course consequences for young people. Yet, we know far less about its burden in lower-income countries. Due to higher fertility and mortality in lower-income countries, the level, timing, intensity, and circumstances surrounding sibling mortality are likely to follow patterns distinct from those in higher-income settings. Thus, in this study, we offer a descriptive overview of sibling death in 43 countries across sub-Saharan Africa, South and Southeast Asia, and Latin America and the Caribbean. Specifically, we analyze Demographic and Health Survey data from nationally representative samples of 352,930 15- to 34-year-old women, born between 1985 and 2003, to document experiences of sibling death before age 25. On average, roughly one-third of individuals report a deceased sibling in these countries; estimates reach 40–50% of respondents in multiple African countries, particularly those that have experienced conflict and war. Although some sibling deaths occurred before the focal respondent was born, most bereaved individuals recalled a death during their lifetime—often in late childhood/early adolescence. High proportions of bereaved respondents report multiple sibling deaths, highlighting the clustering of deaths within families. Even so, bereaved individuals tend to come from large families and thus frequently have a comparable number of surviving siblings as people who never experienced a sibling die. Together, the results offer a window into global inequality in childhood experiences, and they attest to the need for research that explores the implications of sibling mortality for young people in world regions where the experience is concentrated.

## Introduction

Family bereavement is among one of life’s most severe events that can have long-lasting consequences for young people [1, 2]. Although most research on family bereavement focuses on parental and child bereavement, increasing research confirms siblings are also affected by one another’s death.

A sibling’s death can elevate young people’s risk of health problems [3–10] and influence their transition to adulthood [11–13]. Attesting to how influential siblings are [14, 15], a sibling’s death can be more consequential for young people than a parent’s death, at least in terms of its impact on emotional and behavioral problems [16] and health risks [17]. Some studies emphasize that grief from a sibling’s death fades with time, but other studies find that the trauma of experiencing a sibling’s death can act as a prolonged stressor [10].

The growing literature on sibling bereavement, however, is narrow in geographic scope, focusing almost entirely on data from high-income countries in North America and Europe—settings where the sibling mortality burden is, globally speaking, minimal. In the U.S., for example, an estimated 8% of youth experience the death of a sibling before age 25 [12, 18]. In some European populations the prevalence of bereaved siblings is even lower, with a mere 1% of young people having lost a sibling before age 18 in Denmark and Sweden [10].

We lack basic knowledge about sibling mortality in the world’s lower-income, high-mortality countries—the precise settings where the sibling mortality burden is likely far higher due to mortality and fertility patterns. Mortality rates among children and adolescents are substantially higher in most countries in Asia, Latin America, and Africa relative to rates in North America and Europe [19, 20], which likely results in many surviving individuals having experienced a sibling death. However, because child mortality is highly clustered within disadvantaged families in poor countries [20, 21], the extent to which sibling mortality is a diffuse experience, versus one that is highly concentrated within a small proportion of sibling sets, remains unclear.

The higher fertility levels in lower-income countries [22] are also relevant. Although total fertility rates are converging worldwide [23], fertility remains higher in parts of the global south, particularly in Africa [24]. Thus, on top of the higher mortality levels, individuals in lower-income countries tend to have more siblings, which increases their exposure to the risk of having a sibling die. However, it is unclear if the clustering of deaths within larger families results in bereaved individuals experiencing an actual hollowing of their sibling set, and thus fewer adult siblings, or if larger family sizes offset these losses, resulting in bereaved youth having a comparable number of remaining siblings as those never bereaved.

Beyond the need to clarify the overall prevalence and patterns of bereavement from the vantage point of siblings, additional questions about the timing and circumstances of sibling death remain unanswered. For instance, it is unclear if sibling deaths typically occur before individuals are themselves alive; demographic research suggests the death of a preceding sibling may be a key catalyst for an individual’s own birth [25]. Additionally, in contexts of high child mortality, most sibling deaths occur at young ages, when the focal individuals themselves are young and unlikely to have cultivated a strong bond with the sibling. The implications of sibling mortality for women’s future outcomes vary depending on the timing and circumstances surrounding the death [13, 26, 27]. Thus, developing a descriptive overview of how sibling death is typically experienced—from the perspective of surviving siblings—will help clarify its potential ramifications.

In this study, we address the dearth of knowledge on the frequency of, and circumstances surrounding, sibling loss by studying the experiences of a recent cohort of younger women (ages 15–34; born 1985–2003) in 43 countries in sub-Saharan Africa, South and Southeast Asia, and Latin America and the Caribbean. To do so, we use sibling mortality data collected by the Demographic and Health Survey. Numerous studies have carefully leveraged this sibling history data to understand patterns of adult mortality in low-income countries [28–33], which was the intention of their collection. Yet, no studies have sought to offer an experiential understanding of sibling death; thus, this study seeks to identify the contours of sibling loss as remembered by those affected, and how they vary across world regions. By offering a descriptive overview of the frequency of, and circumstances surrounding, sibling mortality, this study offers a guidepost for future work interested in identifying the individual and life course implications of sibling death in the world regions where this experience is concentrated.

## Background

### Significance of sibling death

Research shows that having a sibling die can have wide-ranging effects on individuals’ mental and physical health. In North America and Europe, for example, having a sibling die is associated with an elevated risk of poor psychological outcomes, including complicated grief and severe mental distress [5–9, 34, 35]. However, few studies highlight the possible grief, loss, and trauma associated with sibling bereavement in lower-income countries where fertility remains much higher and life expectancy much lower (see, for example, [36]). Instead, research on traumatic loss in low-income countries has focused mostly on parental loss, particularly in the context of war and natural disasters [36–38].

As a result, we do not know how common sibling bereavement in these settings. Nor do we know if individuals commonly experience sibling deaths at ages when they are old enough to have had a personal, emotional relationship with the deceased. Clarifying the frequency with which individuals lose siblings at ages when they are likely to have a direct relationship with the sibling will shed light on the potential for sibling bereavement to be a contributor to mental health problems in low-income countries. One study of youth in South Africa suggests the premature loss of a sibling is a major risk factor for poor mental health outcomes [39]. With increasing recognition that mental health problems are a key contributor to morbidity in lower-income countries [40], and much of the world’s youth population now coming of age in these countries [41], there is a growing need to identify the prevalence of sibling bereavement, and its potential to act as a psychological risk factor.

It is possible, however, that few individuals experience the death of siblings during their later childhood, adolescence, or young adulthood. The bulk of sibling deaths may occur prior to an individual’s own birth or when they—and their sibling—are in infancy or early childhood. Even though these deaths may not directly affect youth’s mental health, such deaths could still adversely affect youth due to the stress the death places on the siblings’ shared parents. Thus, there is a need to clarify how many individuals are either born to, or spend their early childhood with, bereaved parents. Experiencing a child die is an incredibly stressful experience for parents that can have lasting implications for their physical and psychological well-being and their marriage [42–54]. This is true in not only high-income contexts, but also low-income settings, where child death is a more common, and perhaps even anticipated, element of parenthood [55–58]. Moreover, if a sibling dies from a prolonged illness, the cost of caring for that terminally ill individual may place surviving family members in especially precarious economic situations [59, 60]. These adverse parental outcomes associated with bereavement may spill over to negatively affect the overall physical health and development of the deceased child’s surviving siblings.

Even as the type of health risks associated with sibling death may be contingent on when individuals experienced their sibling die, regardless of timing, a sibling’s death is likely a formidable life experience that shapes individuals’ outlook and perceptions. Exposure to mortality during early life, particularly in one’s own family, can powerfully influence individuals’ perceptions of mortality risk [61, 62]. Even if one does not recall a sibling’s death, and only learns of it from other family members, having premature death be an aspect of one’s family narrative could leave an impression on youths’ perceptions of the likelihood that they or their future children, will prematurely die. Scholars have long hypothesized that these elevated mortality perceptions may influence individuals’ health-related behaviors, risk-taking, and concern for their own children’s survival, which together may help explain the link between sibling mortality and health behaviors [63], fertility ideals [64], and fertility outcomes [26, 65].

Beyond perceptions and health- and fertility-related behaviors, experiencing a sibling die can influence other aspects of youths’ transition to adulthood, including their education [12, 13]. In countries where it is common to lose grandparents and parents before adulthood, sibling interdependencies are often magnified [66, 67]. In such settings, siblings are often a key source of financial stability and support, and they commonly help one another complete school and delay marriage [67]. As such, the death of a sibling means there is one less sibling from whom an individual can draw support, and this assault on a key support system may adversely influence young people’s transition to adulthood in lower-income countries [26, 65, 68, 69].

The adverse social and economic implications of sibling death may be especially relevant in settings where sibling mortality is tightly clustered in a subset families and thus where loss corresponds with a more dramatic hollowing of one’s family support. Patterns of mortality clustering among young children imply that a high proportion of surviving, bereaved individuals will have lost multiple siblings. Even so, individuals who have survived numerous sibling deaths may be from significantly larger families and thus may not have fewer remaining siblings relative to the non-bereaved. That is, because bereaved parents may have additional children in direct response to the death of their child [25, 70], it is not readily apparent that sibling death actually leads to fewer remaining siblings. Clarifying the implications of sibling loss for individuals’ number of remaining siblings will help elucidate the potential for sibling mortality to alter the availability of lateral familial support.

### Studying sibling death with survey data

Evidence that sibling death is highly relevant to individuals’ health and well-being, perceptions, and life course experiences raises questions of how best to estimate the population burden of sibling death. Prospective data collection projects that follow families from initial formation until children age into adulthood would offer the gold standard for tracking family processes such as loss and bereavement. That is, rather than rely on retrospective reports from survivors up to several years later, it would be ideal to have prospective data on families and to track deaths and their possible consequences over time. However, long-term family panel studies are rare, especially in lower-income contexts. Instead, most population research on family member loss relies on cross-sectional surveys that collect retrospective information about prior deaths of family members from surviving individuals.

A drawback of this design is that many people, particularly those of higher birth order, may be unaware of siblings who died before their own birth or died when they were very young. Thus, one could reasonably argue that the most accurate way to estimate the number of bereaved siblings would be to rely on women’s birth histories, which are the basis for tracking child mortality in low-income countries. However, if the primary interest in estimating sibling death is to study the experiential aspect of sibling loss, and to explicitly identify the losses that affect young people’s perceptions, wellbeing, and life experiences, the potential undercounting of sibling deaths due to reliance on surviving siblings’ reports is less concerning. That is, if individuals are not aware of a sibling’s death and have never been made aware of the death by their bereaved relatives, it is difficult to hypothesize how such a death could dramatically influence their life course. Thus, relying on respondents’ direct reports of sibling death can better identify the deaths that live on in the memory of surviving siblings and are more likely to have influenced their lives.

Even so, sibling history data, like women’s reproductive history data, suffer from additional biases. To the extent that mortality is clustered within families, sibling mortality estimates are biased by the selection of survivors: just as children whose mothers died are undercounted in survey research on child mortality [71], a sibling’s death will not be reported if the reporting sibling also died prematurely; thus, estimates based on sibling reports are conservative ones of the total number of individuals who have ever suffered sibling loss. Of course, if sibling deaths are overrepresented in larger families, the overrepresentation of remaining siblings from these larger sibling sets, particularly in a household sampling design, could upwardly bias observations. Additionally, family-based mortality data can suffer from retrospective reporting error. Some individuals will likely fail to recall certain sibling deaths that they were once aware of, especially if the deaths occurred several years prior, or if the siblings were not in close contact [72, 73]. Additionally, there is evidence that siblings who died young are most likely to be undercounted [74]. This could be because individuals were unaware of these deaths or because they are more easily forgotten relative to the deaths of older siblings.

Correcting for these biases is essential when using sibling history data to make broader inferences about mortality patterns in a population [28–30, 33], but these limitations are less threatening when one is trying to draw attention to the possible downstream effects of sibling loss on the surviving population, as is the goal of the current study. Nevertheless, it is important to keep in mind that the results from the current study can only tell us about the mortality regime as remembered by the subset of surviving individuals.

Thus, in this study, we use sibling history data to offer a descriptive overview of sibling mortality in 43 low-income countries in Asia, Africa, and Latin America. First, we estimate the total prevalence of individuals who have experienced a sibling die, and we clarify whether these deaths occurred before birth or during one’s own lifetime and, if so, when. Second, we offer insights into the characteristics of deceased siblings, including their age and gender, to further clarify the types of loss individuals have endured. Finally, we demonstrate the concentration of multiple sibling deaths within families to clarify the implications of sibling mortality on individuals’ remaining number of siblings.

## Data & sample

The Demographic and Health Survey Program (DHS) is a widely used, nationally representative survey program that has collected data in more than 90 low-income countries for more than 30 years (see https://dhsprogram.com/ for more information) [75]. In participating countries, DHS Program surveys are typically administered every five years. The DHS’s household-based surveys feature three core questionnaires: the household questionnaire, the women’s questionnaire, and the men’s questionnaire. In sampled households, DHS interviewers ask a single individual questions about the household and its members. Additionally, all women age 15 to 49 years are eligible to participate in one-on-one interviews. A subsample of men age 15 to 54 (or 59) are also asked to participate in one-on-one interviews. The surveys are standardized to allow for comparisons across countries. Response rates are generally high (>98%). The DHS considers non-response at the household and individual levels when generating sample weights, which we use when calculating estimates shown here.

In several countries, the DHS incorporated a sibling history module into the one-on-one surveys with the primary intention of tracking adult mortality conditions [33]. Because of the difficulty tracking adult mortality in low-income countries that lack civil registration and vital statistics data, this “indirect” survey-based approach has become widely used, having grown out of the “sisterhood method” for studying maternal mortality [76]. The survey module collects information from respondents on each of their siblings born to the same mother, including the sibling’s gender, birth year, and vital status. Note that twin siblings are included in the study and are treated identically to singleton siblings. For deceased siblings, respondents are asked the year the sibling died. In most countries, the DHS only includes this module in the women’s interviews; sibling history data are only available from men in select countries [32]. Given evidence of minimal differences in mortality reporting among men and women [32], here we use sibling mortality data from the women’s surveys only.

Because mortality conditions are evolving rapidly in low-income countries, to offer an overview of sibling mortality among current young adults, we focus on individuals born between 1985 and 2003, who were thus between 15 and 34 years old at the time of the survey. Moreover, because sibling death is highly consequential when experienced at younger ages, we follow past studies and focus on sibling deaths that occurred before an individual was age 25 [12]. This also has the advantage of reducing recall error given the shorter recall period. We limit data to surveys collected since 2007, which include sizeable samples of women who are currently or recently were between the ages of 15 and 25. In all, we leverage sibling mortality data from 44 countries across six lower-income regions, including Western, Central, Eastern, and Southern Africa; Latin America and the Caribbean; and South and South-East Asia. See **S1 Table** for a full list of the countries, survey years, and sample sizes.

Note that this research was approved by the University of Southern California Institutional Review Board (study approval ID: UP-20-00015). The data were analyzed anonymously, hence no consent obtained. All data replication files from the article are posted at: https://doi.org/10.7910/DVN/VBQJFY [77].

## Measures and statistical analysis

### Total percentage of bereaved siblings

Our first set of descriptive analyses offers an overview of the prevalence of individuals who have ***ever had a sibling die***. Using individuals’ reports of their siblings’ vital status at the time of the survey, we first generate a binary indicator of whether the respondent reports having one or more siblings who are no longer alive. Even though some women are older than 25, note that we only include deaths that were reported to have occurred before the respondent’s own 25^th^ birthday. We do, however, include the 2,347 individuals as bereaved who reported a sibling death, however, could not specify when it occurred. (0.67% of the full sample) reported that one of their siblings died, yet they were unaware of when the sibling died.

After presenting the total percentage of individuals who have ever had a sibling die in each country, we next distinguish between individuals who were born to a bereaved mother and those who experienced a sibling die during their own lifetime. To capture the total percentage of respondents who ***had a sibling die before birth***, we use data on the year of the respondent’s birth and the year of the sibling’s death. This indicator clarifies the total percentage of individuals who were born to a bereaved mother. We again use information on the year of the respondent’s birth and the year of the sibling’s death to calculate the percentage of women who ***ever had a sibling die during their lifetime*.** This indicator clarifies the percentage of individuals who experienced the death between age 0 and 25 years old.

### Age at first sibling death and cumulative probability of sibling death over the life course

This first set of indicators offers the cumulative prevalence of sibling bereavement and differentiates between sibling deaths that occurred before and after one’s birth, yet understanding the timing of sibling death during one’s life is critical to theorizing the possible consequences these deaths may bear for an individual. Thus, in a second set of analyses, we focus on sibling deaths that occurred during one’s lifetime, and we clarify the age at first sibling death and the cumulative probability of experiencing a sibling die between birth and age 25.

In these analyses, we create a hazard function where a failure event is defined as the time of the first sibling death between the respondent’s birth and age 25. That is, we restructure the data to represent person-years to model the timing of sibling death between birth and age 25. This approach allows us to address censoring (e.g., not all respondents experienced a sibling die or reached age 25) and to explicitly model time to the first (recalled) sibling death. That is, not all respondents had reached age 25 at the time of the survey, so some may go on to experience a sibling die—a reality that this approach adjusts for accordingly. Moreover, this approach enables us to understand the timing of sibling death. Given that the cumulative hazard estimates the probability of an event based on respondent’s age, in these analyses, all respondents begin the hazard at age 0. Thus, we exclude all events (i.e., sibling deaths) before the focal respondent’s birth from this measure; however, we include respondents who were born to bereaved parents. To present these results, we graph the inverse of the Kaplan-Meier survivor function (i.e., the cumulative distribution function) to demonstrate the ***cumulative probability of sibling death between birth and age 25*.** For parsimony, we aggregate these estimates and present them for the six geographic regions; in supplementary analyses, we also present the country-specific findings (**S1 Fig**).

Because it is difficult to glean the average age of respondents at the time of their sibling’s death from the cumulative probability estimates, in additional analyses, we present the ***average age of respondents at the time of the first sibling death*,** by country. That is, among youth who have ever lost a sibling, we use information on the respondent’s birth year, as well as the year their sibling died, to calculate the mean age of respondents at the time their first sibling died separately for each country. This indicator, in addition to the cumulative probability estimates, offers a descriptive overview of the timing of sibling death and country-level variation therein.

### Characteristics of the deceased sibling

The above estimates offer a detailed overview of the life course timing of individuals’ recalled experience of sibling mortality, however, they do not offer insight into the characteristics of the deceased sibling. Thus, in a next set of analyses, we focus on characteristics of the deceased sibling, including their age and sex. For each deceased sibling, respondents report the sex of the sibling as well as the year the sibling was born and the year the sibling died, allowing us to calculate the age of the sibling at the time of death. With this information, we generate region-specific population pyramids that depict the **age and sex distribution of deceased siblings.** Note that these estimates pertain only to the first sibling death recorded in the prior hazard analysis. We list all country-specific estimates of the percentage of deceased siblings who were female, as well as their average age at time of death, in **S2 Table**. To further clarify the relative position of the bereaved and deceased siblings in their family, in **S2 Table** we tabulate the percentage of deceased siblings who were older (versus the same age or younger) than the focal respondent.

### Intensity of sibling loss and number of remaining siblings

In a last set of analyses, we clarify the extent to which individuals have experienced multiple sibling deaths as well as the consequences of these deaths for their number of remaining siblings. To demonstrate multiple sibling losses, we first calculate the ***total number of deceased siblings*** a respondent reports. This calculation includes siblings who are reported to have died before and after the respondent’s birth, as well as deaths that the respondent were unsure of when they occurred. Using this information, we tabulate the percentage of bereaved siblings who lost one, two, three, or four or more siblings, and we present the results for each country.

We also calculate the **number of remaining siblings** of bereaved (and non-bereaved) respondents, allowing us to clarify the extent to which sibling loss leaves individuals with fewer siblings. All respondents, including those who have never had a sibling die, report their total number of siblings. Among bereaved siblings, we calculate the number of remaining siblings by subtracting the number of siblings reported deceased from the total number of siblings ever reported. These analyses allow us to assess whether sibling death compromises the number of remaining siblings available to individuals or whether bereaved siblings tend to come from larger families and thus have a comparable number of siblings even after experiencing sibling death. Note that to maintain the representativeness of the sample, we include single children among the group who have never experienced a sibling die.

## Results

### Total percentage of bereaved siblings

**Fig 1:**
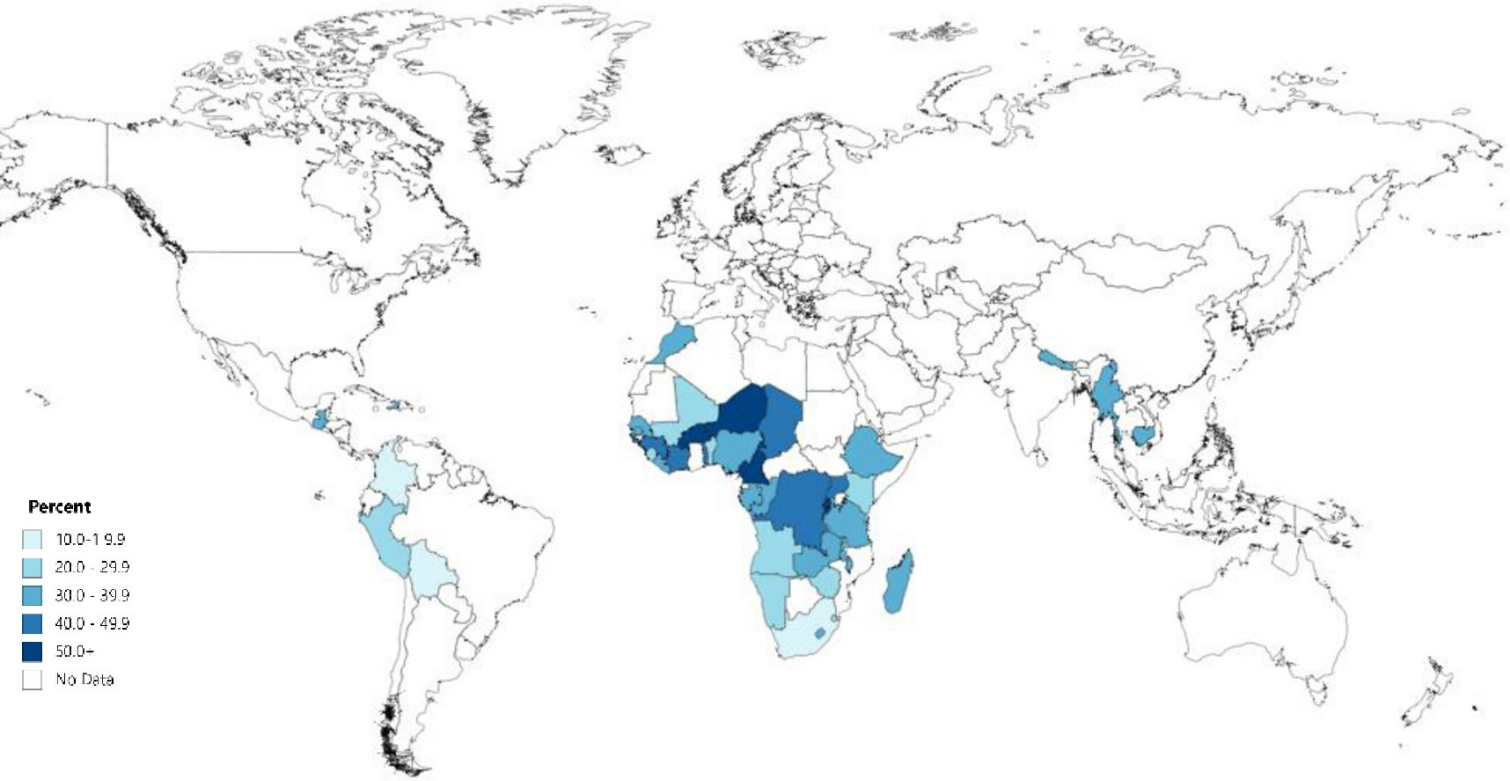
Percentage of young women (15–34) who have at least one deceased sibling before age 25 in 44 countries across Western, Central, Eastern, and Southern Africa, South and Southeast Asia, and Latin America and the Caribbean. Estimates calculated based on Demographic and Health Survey data (https://dhsprogram.com/). All women ages 15–34 at the time of the survey were born after 1984. See S1 Table for a full list of countries and point estimates. Figure created using mapchart.net.

**Fig 1** (and **S1 Table**) offers a comprehensive overview of the percentage of women age 15–34 (born 1985–2003) who have at least one deceased sibling in the 43 countries across Africa, Asia, and Latin America and the Caribbean. As depicted in **Fig 1**, in each of the 43 countries studied here, over 10% of young women have at least one deceased sibling. Across all countries, roughly one-third of respondents reported at least one sibling in their family who is no longer alive—a sibling mortality burden that dwarfs that experienced by youth in the U.S. and Europe.

**Fig 1** also shows important regional variation in the percentage of individuals who have experienced a sibling die. In multiple countries in Western and Central Africa, including Burkina Faso, Democratic Republic of the Congo (DRC), Cameroon, and Niger, more than one-half of respondents have at least one deceased sibling by age 25. The same is true in select Eastern African countries, including Burundi and Rwanda. In several Southern African countries, nearly one-third of respondents have at least one deceased sibling. South Africa is an outlier, with 12% of young women having a deceased sibling, respectively.

Sibling mortality is also widely reported in South and Southeast Asia, with roughly one-third of respondents reporting a deceased sibling in Cambodia, Myanmar, and Nepal. The same is true in Latin America and the Caribbean, particularly in Guatemala and Haiti, where approximately one-third of respondents report a deceased sibling. Sibling loss is relatively less common in Bolivia and Columbia, yet it is still experienced by more than 10% of respondents.

This cumulative indicator includes sibling deaths regardless of when they occurred. That is, these deaths reflect both the experience of being born to a bereaved mother and the experience of having a sibling die during one’s own lifetime, specifically between birth and age 25. Thus, in **S1 Table**, we present estimates for these two distinct types of sibling loss. **S1 Table** shows that both experiences of loss are common, but in most countries, substantially more women report the death of a sibling during their lifetime than a sibling death before they were born. On average, roughly 12% of respondents report being born to a bereaved mother, with as many as 25% reporting a sibling who died during their lifetime.

### Age at first sibling death and cumulative probability of sibling death over the life course

**Fig 2:**
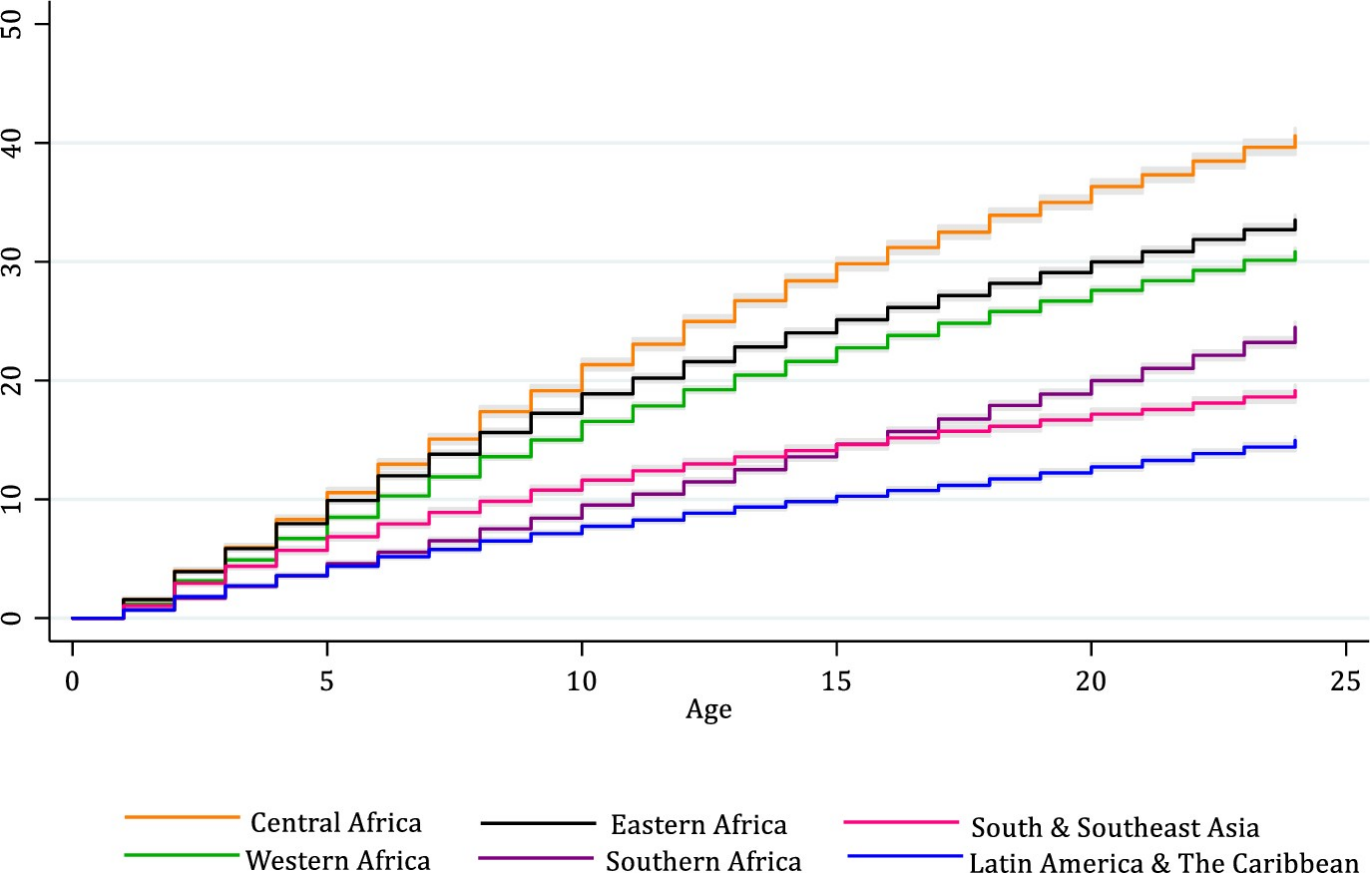
Cumulative probability of experiencing a sibling die between ages 0 and 25 among young women in 44 countries, by world region. Gray represents 95% confidence interval. See S1 Table for full list of countries included in each region. Estimates calculated based on Demographic and Health Survey data (https://dhsprogram.com/).

To further understand the circumstances surrounding the experience of sibling death during one’s lifetime, specifically its timing in the life course and its overall probability before age 25, **Fig 2** presents the Kaplan-Meier estimates of the cumulative probability of experiencing a sibling die between birth and age 25, by region (see **S1 Fig** for country-specific estimates). As shown in **Fig 2,** in Central Africa, nearly 40% of respondents report a sibling death before age 25, with many of these deaths reported to have occurred in later childhood through young adulthood. In Western and Eastern Africa, upward of 30% of respondents have experienced a sibling die. Estimates are lower in the remaining regions, yet even so, between one-in-five and one-in-six respondents report experiencing a sibling die in early life in Southern Africa, South and Southeast Asia, and Latin America and the Caribbean.

In addition to documenting the high cumulative probability of recalled sibling loss, **Fig 2** also clarifies the heterogeneity in the age at which respondents recall experiencing a sibling die. The slope is initially steep but then flattens in Asia, suggesting that more women report having experienced a sibling death at younger ages. The slopes for Central, Western, and Eastern Africa confirm that many women recall having experienced a sibling die early in life, but the probability continues to climb through later childhood, adolescence, and early adulthood. In Southern Africa, fewer women recall a sibling die early in their life, but the probability increases sharply from adolescence through young adulthood.

**Fig 3:**
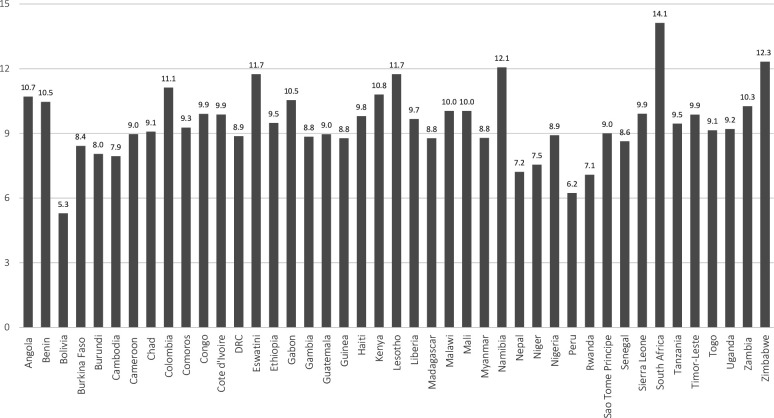
Average age at time of first sibling death, by country. See S1 Table for full list of countries included in each region. Note that, in the case a respondent has experienced multiple siblings die, these estimates pertain to the respondents’ age at the time of the first sibling death. Estimates calculated based on Demographic and Health Survey data (https://dhsprogram.com/).

To further clarify individuals’ age at the time of their sibling’s death, and to document variation between countries, **Fig 3** presents the average age respondents first recall experiencing a sibling die during their lifetime. In several countries, including Bolivia, Nepal, Peru, and Rwanda, bereaved women report first experiencing a sibling die in their early childhood, ranging from an average age of 5 to 8 years old. In several other countries, however, bereaved individuals report being in later childhood, and even into adolescence, when they first experienced a sibling die. For instance, in Eswatini, Lesotho, Namibia, South Africa, and Zimbabwe—all Southern African countries with severe HIV/AIDS epidemics—the average age of bereavement was between 12 and 15 years old. The same is true of Colombia—a country with one of the world’s highest death rates due to violence. Together, these estimates confirm variation in the average age of first sibling bereavement during one’s lifetime, and that many young people in lower-income countries first recall experiencing a sibling die at impressionable ages in later childhood and even adolescence. Of course, research on recall errors associated with the timing of sibling deaths suggests this could be due to individuals’ selective recall of these, perhaps more traumatizing, losses, rather than those that occurred earlier in their childhood.

### Characteristics of the deceased sibling

**Fig 4:**
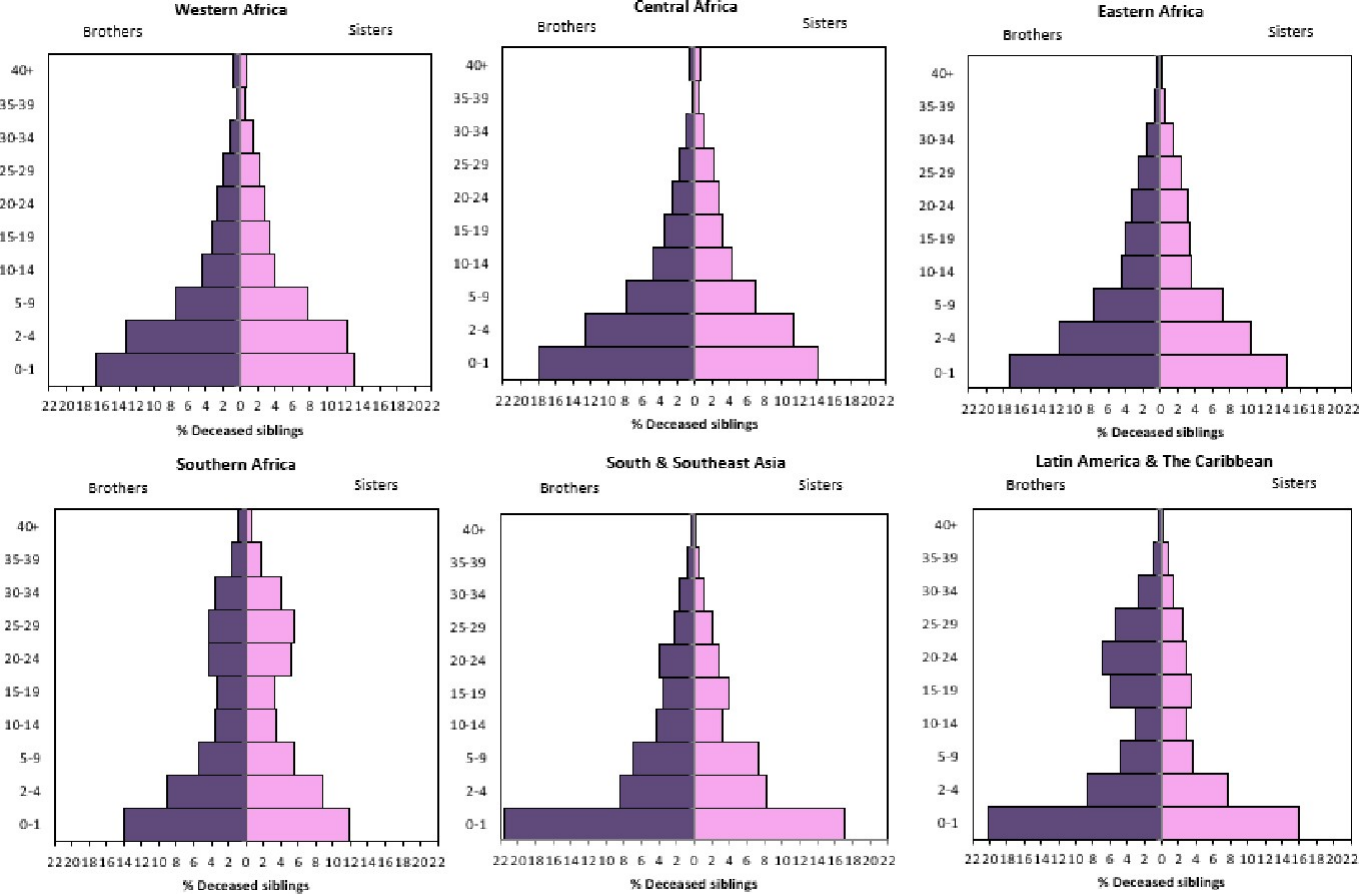
Age and sex distribution of deceased siblings, by region. See S1 Table for full list of countries included in each region, and S2 Table for country specific estimates of the deceased sibling’s age and gender. Note that, in the case a respondent has experienced multiple siblings die, these estimates pertain to the first sibling death. Estimates calculated based on Demographic and Health Survey data (https://dhsprogram.com/).

To offer insights into the characteristics of the deceased siblings on which women report in Figs **2** and **4** depicts region-specific population pyramids of the age and sex distribution of the deceased siblings. As noted, in the case of respondents with multiple deceased siblings, these estimates pertain only to the first sibling they experienced die during their lifetime, between ages 0 and 25 (i.e., the deceased sibling that is the basis of results in Figs **2** and **3**). As shown in **Fig 4**, the age and sex distribution of deceased siblings is highly variable across regions (see **S2 Table** for country-specific estimates). In Western, Central, and Eastern Africa—world regions that continue to have the highest child mortality rates—a high proportion of deceased siblings were brothers and sisters under age five. Yet a high percentage of respondents in these regions also reported having an adolescent, or even adult sibling die. The age and sex distribution of deceased siblings in Southern Africa is notably different, likely due to the intensity of HIV/AIDS epidemics in these countries that have relatively lower under-five mortality rates. Although many individuals report experiencing the death of a young brother or sister, a high percentage of deceased siblings—specifically sisters—are reported to have died between the ages of 20 and 29.

**Fig 4** shows that in South and Southeast Asia, respondents report a higher proportion of deceased siblings died in infancy, with a notable overrepresentation of infant brothers reported relative to infant sisters. Many respondents in Latin American and Caribbean countries also reported siblings who died in infancy, and again there is a higher proportion of deceased infant brothers relative to sisters, possibly attributable to these reports stemming from women. However, like Southern Africa, in Latin America and the Caribbean, there is a clear bimodal age distribution of deceased siblings, with respondents reporting a higher concentration of siblings who died between ages 20 and 29. Individuals are unlikely to perfectly report on the timing of a sibling’s birth and death, and thus these estimates will be biased accordingly. However, the regional variation—which maps on to regional differences in mortality—are suggestive of true differences in the actual experience of sibling loss across these settings.

**S2 Table** provides country-specific estimates of the age and sex of deceased siblings. Additionally, **S2 Table** further clarifies the relative age and position in the family of the deceased and bereaved siblings. In line with the age distributions presented in **Figs 3** and **4**, in several Western African and South and Southeastern Asian countries, a lower percentage—as few as one-third—of bereaved individuals report first experiencing an older sibling die. Conversely, in several Latin American and Southern African countries, most bereaved individuals report first experiencing an older sibling die.

The relative and absolute age of deceased siblings, and the notable variation across world regions, raise further questions of whether the likelihood of reporting a sibling death in one’s lifetime is related to individuals’ birth position in their family. Thus, in supplementary analyses (see **S2 Fig**), we explored whether individuals’ position in the family as a first, middle, or lastborn child influenced their likelihood of reporting a deceased sibling—either one that died before their birth (left panel of **S2 Fig**) or during their lifetime (right panel of **S2 Fig**). As shown in **S2 Fig**, respondents’ position in the birth order—either as a middle or last child—does not dramatically influence their likelihood of reporting having been born to a bereaved mother, suggesting that middle and lastborn children are equally likely to be aware of an earlier-born sibling who died prior to their own birth.

In terms of experiencing a sibling death in one’s lifetime, however, position in one’s family does influence who reports a deceased sibling—and thus helps illuminate the patterns revealed by prior analyses. As shown in the right panel of **S2 Fig**, in most regions, lastborn children are the least likely to report that a sibling died during their lifetime, and thus they are the least likely to contribute to the results in Figs **2–4**, which may contribute to the timing patterns and characteristics of the deaths captured here. In Southern Africa, however, we found the opposite to be true: lastborn children are more likely to report having a sibling die during their lifetime relative to firstborn children. This is likely due to the higher concentration of deaths of older siblings in the region, resulting in even lastborn children being affected by sibling death. In all six regions, middle children are the most likely to report a sibling death during their lifetime, likely an artifact of their higher exposure to having both older and younger siblings die. These findings offer an important reminder that while mortality and mortality exposure are undoubtedly socially patterned, the types of sibling losses captured by bereaved siblings of a particular age are also mechanistic—determined by birth order and current age.

### Intensity of sibling loss and number of remaining siblings

**Fig 5:**
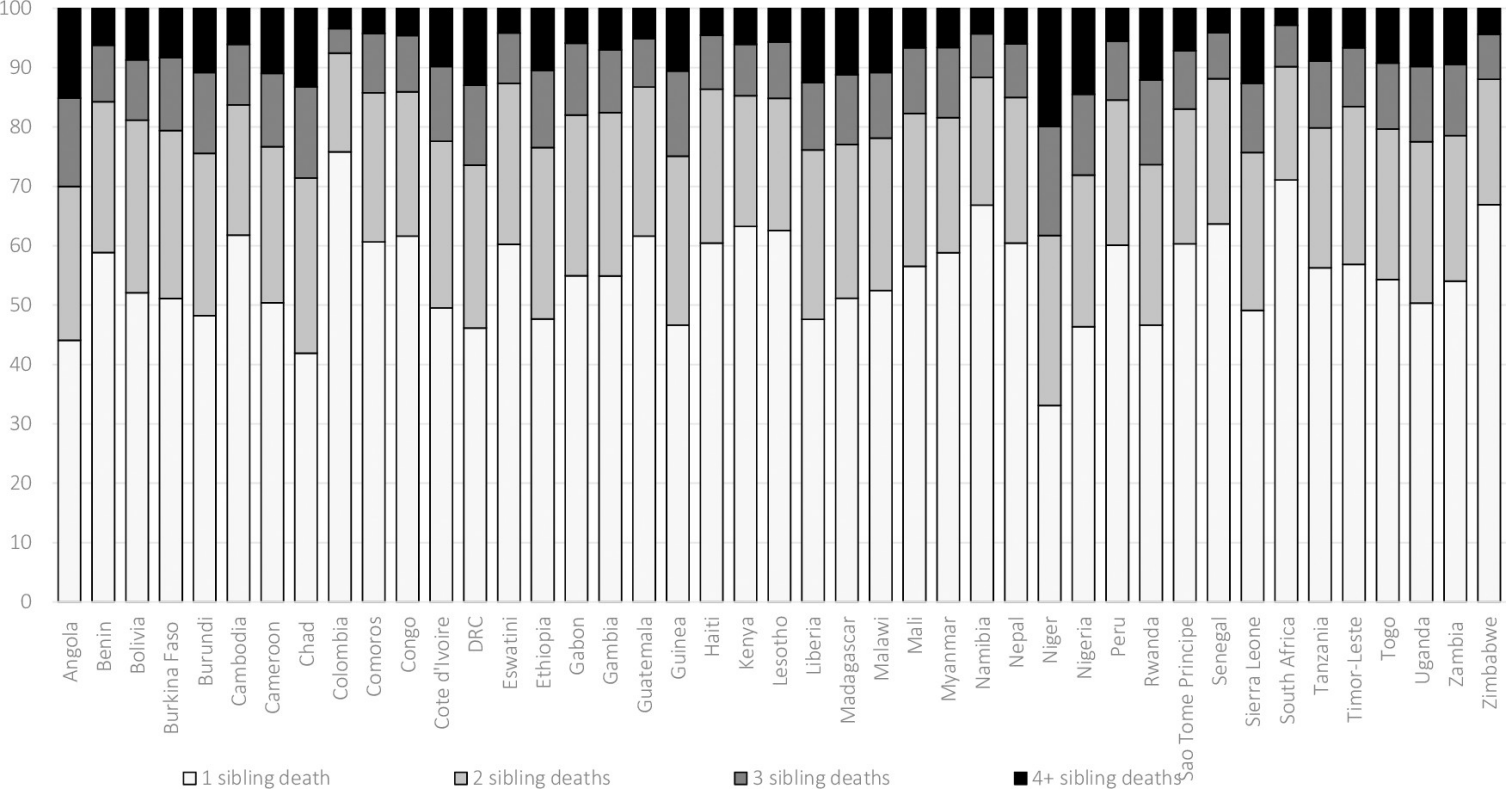
Estimates of the percentage of bereaved siblings who have lost 1, 2, 3, and 4 or more siblings. Estimates calculated based on Demographic and Health Survey data (https://dhsprogram.com/).

Together, these estimates offer insights into the overall prevalence of sibling loss and the experience of sibling death during one’s lifetime. However, these estimates offer no insight into subsequent losses—that is, the potential that respondents experience multiple siblings die. **Fig 5** presents the total proportion of bereaved individuals who have lost one, two, three, or four or more siblings. Across all countries, the results show considerable mortality clustering within single sets of siblings. In several countries, roughly one-half of bereaved individuals have experienced the death of two or more siblings. In several countries, as many as one-in-five bereaved individuals have experienced the death of three or more siblings. In Asia and Latin America and the Caribbean, upward of one-third of bereaved individuals have lost two or more siblings, attesting to the high level of sibling mortality clustering within families. Multiple sibling losses are even more common in Western, Central, and Eastern African countries, contexts with high fertility and high rates of under-five mortality. Again, it is important to note that these estimates are likely conservative, given selection bias and the omission of deceased individuals.

**Fig 6:**
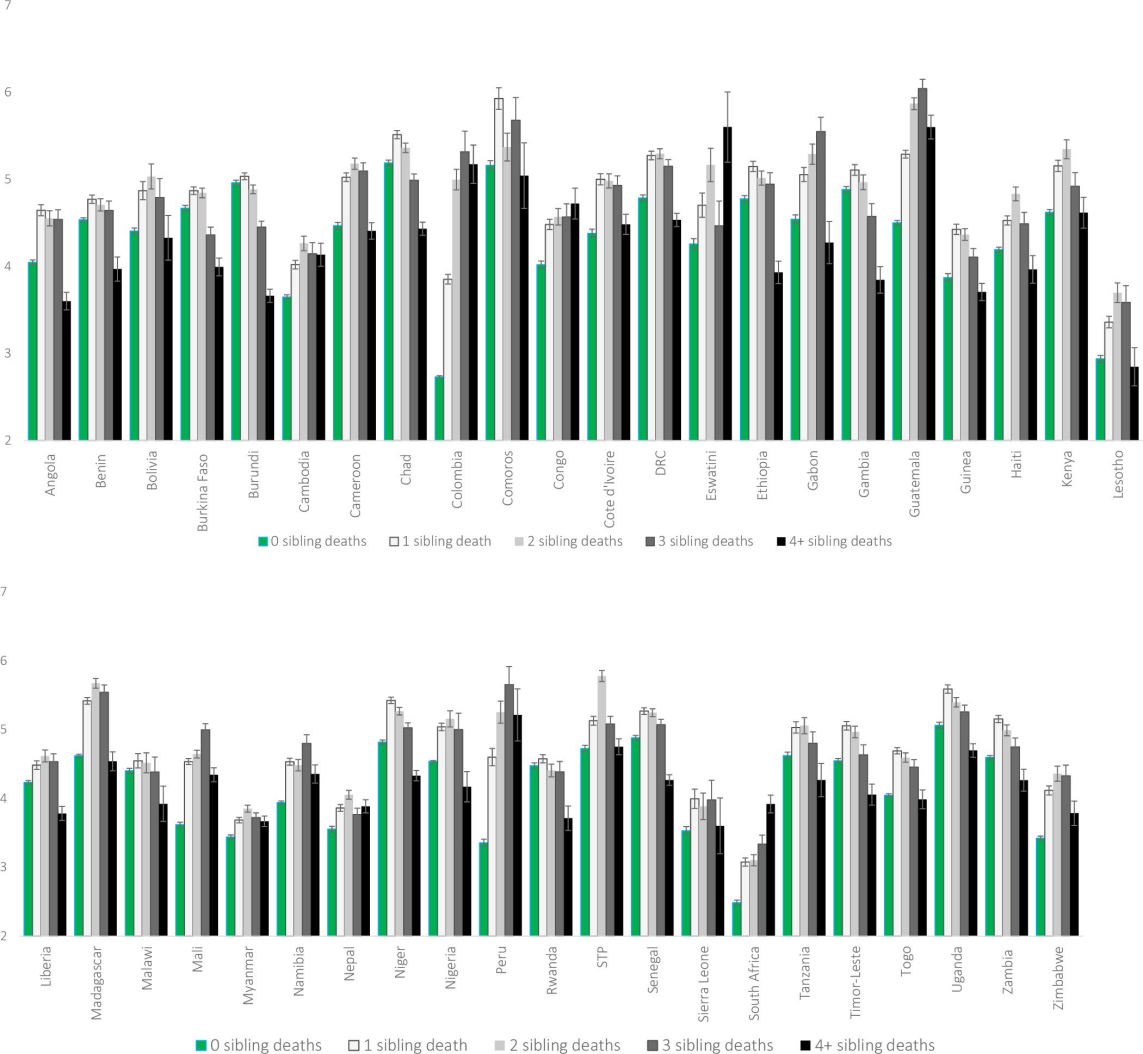
Remaining number of siblings, by history of sibling loss and country.

The high percentage of respondents who have experienced the death of multiple siblings raises questions of whether these individuals have, on average, significantly fewer remaining siblings than do non-bereaved individuals. **Fig 6** compares the average number of remaining siblings according to the total number of deceased siblings (ranging from 0 to 4+). Bereaved respondents—including those who experienced numerous sibling deaths—do not, on average, have fewer living siblings than respondents who never experienced a sibling death. Instead, in most countries, respondents who report having lost one, two, or even three siblings have a comparable number of remaining living siblings relative to their peers whose siblings are all still alive. Only those who report four or more sibling deaths have fewer siblings than non-bereaved respondents, on average. However, this pattern is not observed in all countries. And where it is observed, even respondents with four or more deceased siblings often have, at most, one fewer remaining sibling relative to their peers with no deceased siblings. Additional analyses confirm that only a small fraction of respondents have experienced all their siblings die. Across all countries, estimates range between 1–2% of bereaved individuals having no remaining siblings. The prevalence of bereaved individuals left with no siblings is slightly higher—between 3 and 6%—in select Southern African and Latin American countries (e.g., Lesotho, South Africa, Zimbabwe).

## Discussion

This study offers the first estimates of sibling mortality experiences during early life in 43 countries in six lower-income regions. Even as numerous studies have leveraged sibling history data to understand patterns of adult mortality in low-income countries [28–33], we know little about the contours of the experience of sibling loss. Thus, in this study, we offer a detailed overview of sibling mortality before age 25—as experienced by the surviving sibling—in six world regions across the Global South.

The results show the exceptionally high, although notably variable, levels of sibling loss in lower-income countries. Some of these deaths occur before the focal individual’s birth: upward of 10% of women report being born to a bereaved mother, with estimates ranging from 2–4% (Colombia, South Africa) to 25% (Niger). Prior work shows that the death of a child is a major life course event that fundamentally shapes women’s lives [78]; future research that explicitly studies how bereavement affects parents, and their subsequent children is sorely needed. The particularly high levels of youth born to bereaved mothers in several African countries affected by conflict and war demonstrate the specific need for such research in these settings. These estimates offer a reminder that the deaths accumulated during these crises live on in families’ experiences for years to come and may affect even those who were not yet born at the time of the crisis. Of course, these results also highlight the potential for dramatic cohort differences in youth’s likelihood of having bereaved parents in countries that have recently experienced major mortality shocks; it is imperative to keep in mind that these estimates are specific to women age 15–34 at the time of the survey.

Apart from being born to a bereaved mother, even more individuals report having experienced a sibling die firsthand: the cumulative probabilities of experiencing a sibling die in low-income countries dwarf comparable estimates in high-income ones [10, 12]. Respondents often reported first experiencing a sibling die when they were very young, and thus they were unlikely to have had a direct relationship with the deceased sibling; however, a notable percentage of young women reported first experiencing a sibling die when they were at impressionable stages of childhood, adolescence, and even adulthood. These estimates attest to the need for greater recognition that sibling mortality is an incredibly common source of family trauma for younger women living in low-income countries and one that merits further investigation as a determinant of their future mental health and transition to adulthood. Social science research has long acknowledged that early life experiences have long-lasting consequences for individuals [79, 80]; thus, studies should more explicitly acknowledge the loss of siblings, and other key relatives, as a potentially life-altering experience.

The regional as well as country variation in the circumstances surrounding reported sibling deaths highlights the need for careful, context-specific work on the possible ramifications of sibling death for young people. For instance, in South and Southeast Asian countries, although male infants die at slightly higher rates than female infants, individuals report significantly more deaths among infant brothers relative to infant sisters. This could be a function of the female perspective adopted in this study. Alternatively, this may be tied to son preference [81], and in turn, parents memorializing deceased sons to a greater extent than daughters. That is, the deaths of brothers and sisters may be remembered, recorded, and reacted to in dissimilar ways in South Asia. Conversely, in Southern Africa, bereaved youth disproportionately report sisters having died in their 20s, potentially due to HIV/AIDS and maternal deaths [82]. And in Latin America and the Caribbean, deaths of older brothers are commonly reported, potentially owing to excess male mortality from unintentional deaths, including accidents (e.g., road traffic accidents, occupational hazards) and homicides [83]. Together, these regional age and sex variations in the characteristics of reported deaths highlight the need for future research to carefully attend to possible between-country differences in the interpretation, experience, and repercussions of sibling mortality.

The study further shows how the clustering of deaths is an important aspect of a population’s mortality regime that influences the extent to which the surviving population has experienced multiple siblings die. In several countries, many individuals experience the premature death of multiple siblings while others escape childhood unscathed (or, at least, unaware of sibling death). Results further show that sibling mortality is highly concentrated in larger families. Of course, this association is likely bidirectional: larger families correspond with an increased risk of child mortality [84], and child mortality can lead parents to have more children [70]. Nonetheless, the commonality of having multiple deceased siblings raises questions of the differential effect of experiencing repeated losses versus a single sibling loss. It is reasonable to assume that experiencing numerous mortality events in one’s sibling network will shape young women’s perceptions and behaviors in fundamentally different ways than experiencing a single sibling die. However, importantly, we do not find that respondents who report multiple sibling deaths always have fewer remaining siblings relative to their peers. That is, even as sibling deaths leave holes in individuals’ family systems—holes that will likely alter family dynamics and individuals’ health, behaviors, and outcomes—bereaved individuals mostly come from larger families and thus still have a comparable number of remaining siblings as those who are not bereaved. Thus, studies of the effects of sibling mortality in these countries should recognize that the overall loss of sibling support may not be a prime mechanism by which sibling death influences individuals’ lives.

Together, these results not only offer insights into the typical patterns of sibling mortality in lower-income countries, but they also highlight the tremendous heterogeneity in the experience of sibling death—variation that is visible between and within countries and regions. There is notable variation in the timing and intensity of sibling loss, the characteristics of the deceased sibling, and the effect sibling death has on young women’s remaining family size. Increasingly, research recognizes that the effects of sibling death on individual youth outcomes can vary greatly according to the specific circumstances of the death [13, 26, 27]. Given the very high percentage of women who report some personal experience of sibling death in countries across sub-Saharan Africa, South and Southeast Asia, and Latin America and the Caribbean, there is a need to carefully interrogate these variable experiences to generate nuanced conceptual frameworks of how specific types of sibling loss correspond with young people’s mental and physical health, behaviors, and outcomes.

Even as these findings offer an experiential look at sibling loss in low-income countries, it is important to reiterate that all estimates reported represent only the deaths that the survivors were aware of and readily remembered. Like all retrospective family history data, the information individuals provide about their siblings is imperfect [29]. However, minor shifts in collecting information on siblings has been shown to substantially improve the completeness of reports and accuracy of dates and ages [85]. By recognizing sibling loss as a frequent yet varied early life course experience that can fundamentally alter individuals’ lives, this study highlights the need to further invest in innovative efforts to collect accurate data on sibling loss to facilitate research on how it affects the lives of those who endure it.

## Supporting information

S1 Table List of countries, surveys, and corresponding samples and estimates. (PDF)

S2 Table Additional characteristics about the deceased sibling. (PDF)

S1 Fig Cumulative probability of experiencing a sibling die between ages 0 and 25 among young women in 43 countries, by world region and country. (PDF)

S2 Fig Percent of respondents born to bereaved mother (left panel) and percent of respondents who ever had a sibling die (right panel), by world region and country. (PDF)
