# Supplementary material for: Sibling mortality burden in low-income countries: A descriptive analysis of sibling death in Africa, Asia, and Latin America and the Caribbean
Source: PLoS One. 2020 Oct 14;15(10):e0236498. doi: 10.1371/journal.pone.0236498 (PMC7556453; doi:10.1371/journal.pone.0236498)
Supplement: S1 Table — (PDF) [file pone.0236498.s001.pdf]

SI Table. List of countries, surveys, and corresponding samples and estimates

	Year of survey	Analytic sample size of women 15-34 yrs old	Total % ever had at least one sibling die	% Born to bereaved mother	% Had sibling die during lifetime
**Western Africa**					
Benin	2017-18	10,923	25.4	8.3	20.8
Burkina Faso	2010	7,339	50.4	20.6	38.7
Cameroon	2011	7,921	52.0	21.1	40.9
Cote d'Ivoire	2011-12	5,093	44.7	17.2	35.0
Gambia	2013	6,054	31.6	13.8	23.7
Guinea	2012	4,611	48.3	20.3	38.8
Liberia	2013	4,803	33.6	13.5	26.2
Mali	2012-13	5,346	21.3	5.5	16.5
Niger	2012	5,460	61.7	27.6	51.4
Nigeria	2013	20,731	33.3	12.6	27.0
Senegal	2017	11,536	33.1	14.3	23.7
Sierra Leone	2013	9,131	28.8	9.1	24.2
Togo	2013-14	4,889	40.4	17.0	30.4
**Central Africa**					
Chad	2014-15	11,107	48.1	14.8	42.3
Congo	2011-12	4,782	39.4	12.0	31.7
DRC*	2013-14	10,679	48.5	16.8	41.3
Gabon	2012	4,215	35.1	11.9	25.1
Sao Tome Principe	2008-09	907	40.5	14.6	29.3
**Eastern Africa**					
Burundi	2016-17	11,410	53.1	22.7	41.5
Comoros	2012	2,834	26.1	11.8	16.1
Ethiopia	2016+	10,497	39.7	14.5	28.4
Kenya	2014	8,260	24.9	7.6	20.4
Madagascar	2008-09	6,803	34.3	12.5	27.0
Rwanda	2014-15	7,853	54.9	23.1	43.7
Tanzania	2015-16	8,031	35.6	14.3	27.9
Uganda	2016	12,286	49.0	18.7	35.7
**Southern Africa**					
Angola	2015-16	9,624	28.1	7.2	17.1
Eswatini	2006-07	1,771	26.9	6.7	23.2
Lesotho	2014	3,914	31.8	11.0	25.7
Malawi	2015-16	15,458	33.3	11.2	25.6
Namibia	2013	4,799	22.3	5.8	18.6

South Africa	2016	4,825	12.6	2.5	11.0
Zambia	2013-14	9,194	33.3	10.2	28.3
Zimbabwe	2015	5,911	26.2	6.1	23.0
**South and southeast Asia**					
Cambodia	2014	8,910	30.3	15.1	19.8
Myanmar	2015-16	6,325	30.6	13.6	21.8
Nepal	2016	8,811	36.3	21.2	20.5
Timor-Leste	2016	7,827	17.6	7.6	12.6
**Latin America & the Caribbean**					
Bolivia	2008	5,801	16.5	9.6	7.9
Colombia	2015-16	21,616	11.1	3.6	6.6
Guatemala	2014-15	15,132	33.1	18.1	20.0
Haiti	2016-17	9,419	30.5	12.8	22.0
Peru	2012	10,092	20.6	13.6	9.7

Source: Demographic and Health Survey, *Democratic Republic of the Congo

+Gregorian calendar; data collected in 2008 according to the Ethiopian calendar
