# Supplementary material for: Sibling mortality burden in low-income countries: A descriptive analysis of sibling death in Africa, Asia, and Latin America and the Caribbean
Source: PLoS One. 2020 Oct 14;15(10):e0236498. doi: 10.1371/journal.pone.0236498 (PMC7556453; doi:10.1371/journal.pone.0236498)
Supplement: S2 Table — (PDF) [file pone.0236498.s002.pdf]

S2 Table 2. Additional characteristics of deceased siblings°

	Sample size of deceased sibling°	% Deceased sibling female	Siblings age at time of death		% Deceased sibling older than focal respondent
			Mean	SD	
**Western Africa**					
Benin	2,244	46.4	10.5	6.7	36.3
Burkina Faso	2,797	47.6	8.4	5.6	28.1
Cameroon	3,201	48.1	9.0	6.0	35.8
Cote d'Ivoire	1,850	48.9	9.9	6.1	35.1
Gambia	1,437	46.1	8.8	6.2	34.5
Guinea	1,809	44.9	8.8	5.8	32.1
Liberia	1,354	47.7	9.7	6.4	38.7
Mali	843	49.5	10.0	6.6	40.3
Niger	2,580	49.6	7.5	5.6	32.7
Nigeria	5,629	49.4	8.9	6.2	34.4
Senegal	2,737	46.1	8.6	6.1	37.6
Sierra Leone	2,157	50.5	9.9	6.3	39.8
Togo	1,517	47.2	9.1	6.2	40.5
**Central Africa**					
Chad	4,500	45.9	9.1	6.1	31.5
Congo	1,612	49.2	9.9	6.2	38.8
DRC*	4,461	45.6	8.9	6.0	33.7
Gabon	1,136	48.5	10.5	6.5	45.2
Sao Tome Principe	293	48.7	9.0	5.9	34.1
**Eastern Africa**					
Burundi	4,454	45.3	8.0	5.9	37.4
Comoros	446	46.7	9.3	6.4	37.1
Ethiopia	2,995	47.0	9.5	6.4	41.6
Kenya	1,730	48.2	9.5	6.7	50.9
Madagascar	1,798	48.9	8.8	5.7	36.2
Rwanda	3,350	44.6	7.1	5.3	41.5
Tanzania	2,227	49.3	9.5	6.5	38.8
Uganda	4,497	46.4	9.2	6.4	35.2
**Southern Africa**					
Angola	1,425	47.2	10.7	6.9	37.8
Eswatini	406	54.8	11.7	5.2	56.8

Lesotho	986	49.2	11.7	6.6	56.2
Malawi	3,754	50.3	10.0	6.6	40.9
Namibia	871	48.5	12.1	6.4	56.5
South Africa	582	49.2	14.1	6.7	69.9
Zambia	2,611	53.4	10.3	6.6	48.2
Zimbabwe	1,287	51.9	12.3	6.2	54.7
**South and southeast Asia**					
Cambodia	1,669	44.3	7.9	6.0	40.8
Myanmar	1,465	44.1	8.8	6.5	40.8
Nepal	1,838	49.8	7.2	5.9	32.5
Timor-Leste	893	43.1	9.9	7.0	38.5
**Latin America & the Caribbean**					
Bolivia	394	44.6	5.3	4.0	26.1
Colombia	1,606	29.7	11.1	7.0	55.7
Guatemala	3,047	41.1	9.0	6.4	35.1
Haiti	2,135	46.7	9.8	6.6	44.2
Peru+	1,133	46.6	6.2	5.2	--

Source: Demographic and Health Survey

°References first sibling deceased during respondents' lifetime; *Democratic Republic of the Congo; + DHS Peru data do not feature in information on the year of birth of deceased siblings, but instead only the age at death, hence our inability to calculate the relative age of siblings to focal respondents
