# Supplementary material for: Sibling mortality burden in low-income countries: A descriptive analysis of sibling death in Africa, Asia, and Latin America and the Caribbean
Source: PLoS One. 2020 Oct 14;15(10):e0236498. doi: 10.1371/journal.pone.0236498 (PMC7556453; doi:10.1371/journal.pone.0236498)

S1 Figure . **Cumulative probability of experiencing a sibling die between ages 0 and 25 among young women in 43 countries, by world region and country**

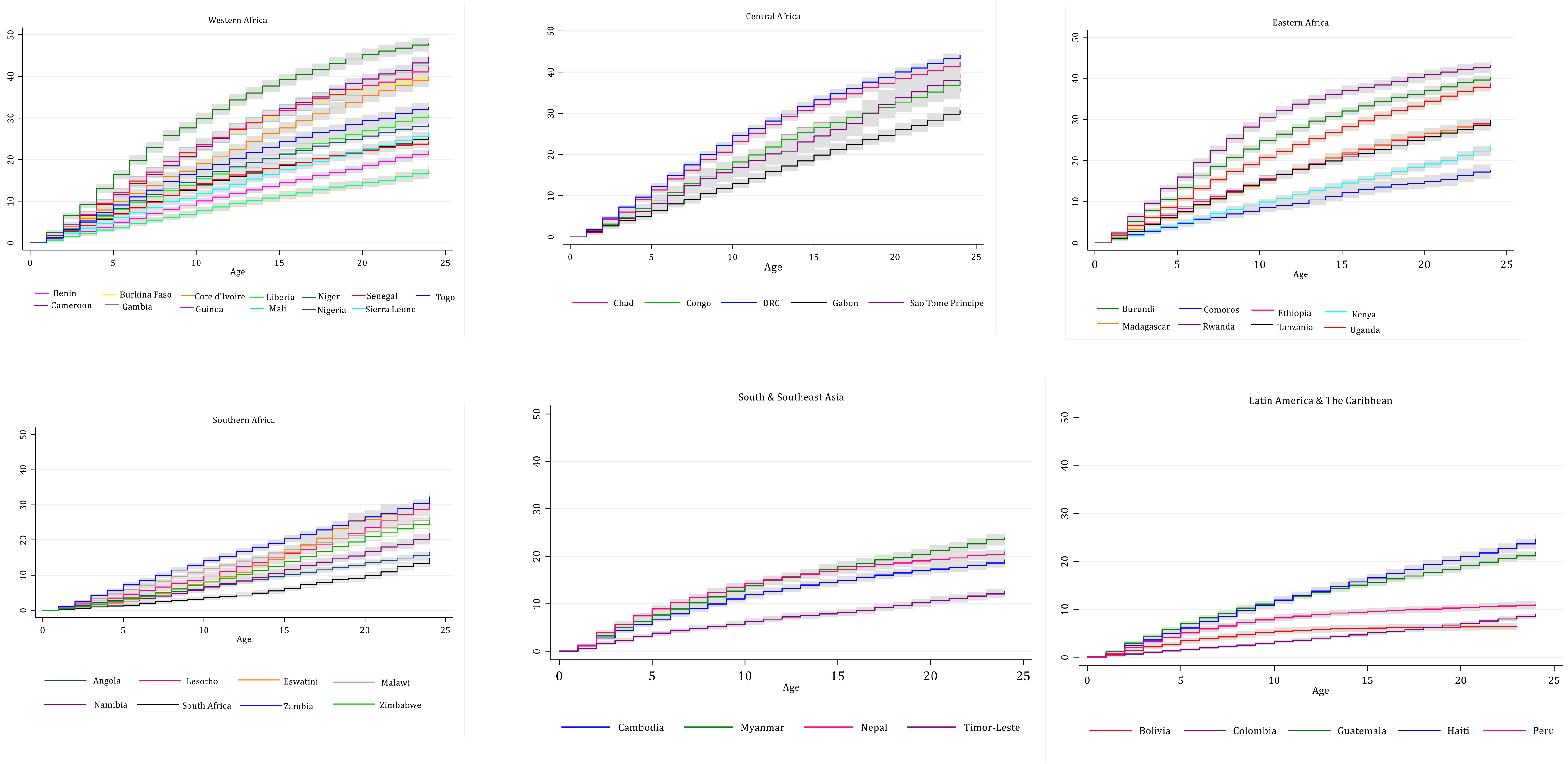

Supplement: S1 Fig — (PDF) [file pone.0236498.s003.pdf]
