# Supplementary material for: Sibling mortality burden in low-income countries: A descriptive analysis of sibling death in Africa, Asia, and Latin America and the Caribbean
Source: PLoS One. 2020 Oct 14;15(10):e0236498. doi: 10.1371/journal.pone.0236498 (PMC7556453; doi:10.1371/journal.pone.0236498)
Supplement: S2 Fig — (PDF) [file pone.0236498.s004.pdf]

S2 Figure. Percent of respondents born to bereaved mother (left panel) and percent of respondents who had a sibling die during their lifetime (right panel), by world region and birth order

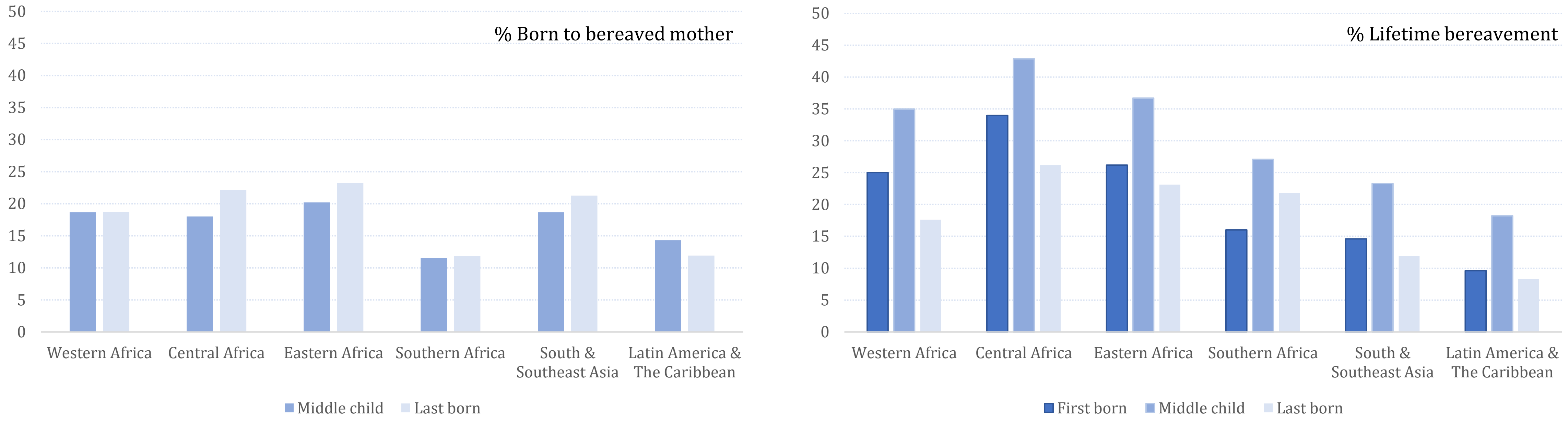

Source: Demographic and Health Survey
